# A Novel Nonsense Mutation of *PHF6* in a Female with Extended Phenotypes of Borjeson-Forssman-Lehmann Syndrome

**DOI:** 10.4274/jcrpe.galenos.2019.2018.0220

**Published:** 2019-11-22

**Authors:** Xia Zhang, Yanjie Fan, Xiaomin Liu, Ming-Ang Zhu, Yu Sun, Hui Yan, Yunjuan He, Xiantao Ye, Xuefan Gu, Yongguo Yu

**Affiliations:** 1 Shanghai Jiao Tong University School of Medicine, Xinhua Hospital, Shanghai Institute for Pediatric Research, Department of Pediatric Endocrinology and Genetics, Shanghai, China; 2 Shanghai Jiaotong University School of Medicine, Pediatric Translational Medicine Institute, Shanghai Children’s Medical Center, The Laboratory of Pediatric Infectious Diseases, Shanghai, China; ★ These authors contributed equally to this work.

**Keywords:** Borjeson-Forssman-Lehmann syndrome, PHF6, X-inactivation, growth hormone deficiency, rhGH treatment, hypogonadism

## Abstract

Borjeson-Forssman-Lehmann syndrome (BFLS) is a rare X-linked disease caused by *PHF6* mutations. Classic BFLS has been associated with intellectual disability (ID), developmental delay (DD), obesity, epilepsy, typical facial features and anomalies of fingers and toes. Endocrinological phenotypes and outcome of treatment in this condition remain to be delineated. Here we report a patient who exhibited complete growth hormone deficiency who responded to hormonal treatment but with adverse effects. Horseshoe kidney was present in this patient, which is also atypical in BFLS. A heterozygous nonsense mutation c.673C>T (p.R225X) of *PHF6* gene was identified in the patient, inherited from her unaffected mother. Both the patient and her mother showed highly skewed X-inactivation. We reviewed the phenotypes of all reported BFLS cases, and summarized their endocrine presentations. This first report of an Asian patient with BFLS further delineated the genetic and phenotypic spectrum of the syndrome. The adverse effect experienced by the patient suggests caution in the use of growth hormone treatment in this condition.

**What is already known on this topic?**

Borjeson-Forssman-Lehmann syndrome (BFLS) is a rare X-linked disease caused by *PHF6* mutations. The features of classic BFLS include intellectual disability, developmental delay, obesity, epilepsy, characteristic face and anomalies of fingers and toes. Endocrine deregulation in BFLS has been reported but not well delineated.

**What this study adds?**

We report a female with a novel nonsense mutation c.673C>T (p.R225X) in the *PHF6* gene. She exhibited certain features beyond the classic BFLS, including complete deficiency of growth hormone and a horseshoe kidney. Adverse effects were elicited after growth hormone treatment in this patient, which has not been previously reported and suggests caution in the use of growth hormone in this condition. We also reviewed all the BFLS case reports and summarized data on their endocrine presentations and treatment.

## Introduction

Borjeson-Forssman-Lehmann syndrome (BFLS), first described in 1962, is a rare X-linked disease (1). So far, about 33 families or sporadic cases have been reported, with 64 patients total (2,3,4). It is characterized by moderate to severe intellectual disability (ID), developmental delay (DD), obesity, epilepsy, hypogonadism, characteristic face and anomalies of fingers and toes (5). This X-linked condition usually affects males, but mild to severe symptoms are present in female carriers and most of them have highly skewed X-inactivation (6). In 2002, Lower et al (7) identified *plant homeodomain finger 6 (PHF6) *as the causal gene of BFLS. Since then, 29 different mutations have been reported in *PHF6* and, among these, 14 mutations were identified in affected females (2,3,4,8,9,10,11,12). All the patients and variants identified were of European ethnicity. In addition, 27 of the BFLS patients were reported to have endocrine abnormalities (10,13,14,15,16,17,18,19,20,21,22,23,24,25). These hormonal abnormalities have not been well summarized so far.

Here we report a Chinese female with a nonsense mutation in the *PHF6* gene, inherited from her mother. Following a thorough review of all reported BFLS cases, we identified some features in this patient beyond those typical of BFLS. In addition, the endocrine aspect of BFLS patients were reviewed and summarized for the first time in the relevant literature. The genetic and phenotypic spectrum of BFLS is discussed.

## Case Report

**Figure 1:**
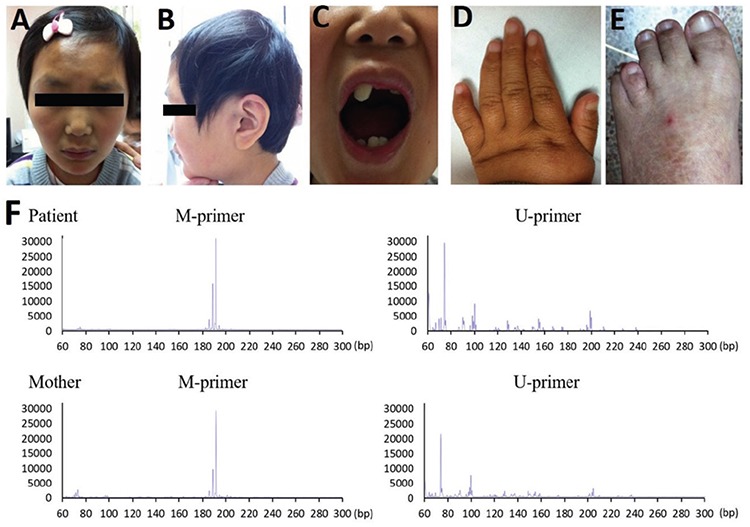
A, B, C, D, E, F) Pictures of our patient at age 9 years. A, B) Facial characteristics, C) Dental abnormalities, D, E) Hand and foot of the patient. The fifth fingers are short and curved, and the fourth toes are short. F) Results of the methylation-specific polymerase chain reaction assay. The inactivated X-chromosome sequence was amplified by the M-primer, the activated X-chromosome sequence was amplified by the U-primer. The result indicated a highly skewed X-inactivation in the patient (98:2) and in her mother (95:5)

A 9 year 1 month old girl presented to the Genetic Endocrinology Clinic with complaints of ID and short stature. Her height was 123 cm [-2 standard deviation (SD)]; height age was 7 years. Her weight was 23 kg (-1.3 SD), and body mass index was 15.2 (25^th^-50^th^ percentile). The height of her father and mother were 168 cm and 157 cm respectively, and the familial target height of the patient was 156 cm (-0.85 SD). She was born by caesarean section post-term, with no history of asphyxia. Her birth weight and body length were 4.25 kg and 50 cm respectively. Severe DD was noticed at toddler stage; she walked alone at the age of three years and could speak a few simple words at the age of five years. She presented with the typical facial features of BFLS, including coarse face, sparse hair, narrow forehead, ptosis, deep-set eyes, broad nasal tip, short nose, malformed teeth and large ears with earlobes of moderate size (Figure 1A, 1B, 1C). She had tapering fingers and fifth curved fingers bilaterally (Figure 1D). She also had flat feet and the fourth toes were shorter than the fifth (Figure 1E). Extensive hyperpigmentation was observed all over the body, but especially on the lower limbs. No secondary sexual characteristics were present at the time of examination. Breast and pubic hair were at stage B1 and P1 respectively (according to the Tanner scale).

Thyroid and liver function tests revealed normal results, but she suffered from a complete deficiency of growth hormone (see Table 1). Her stature was below the 3^rd^ percentile and her bone age was 7^10/12^ years. Due to the complete lack of growth hormone, recombinant human growth hormone (rhGH) injections were commenced at a dose of 0.036 mg/kg/day. However, after three weeks she developed edema in both lower extremities, and the hormonal treatment was ended.

Ultrasonography showed that she had fused kidneys at the lower end (horseshoe kidney). Brain MRI revealed periventricular leukomalacia and hyaline compartment formation. The pituitary appeared thinner than girls of the same age, though definitive measurement of the pituitary size was not performed. Her karyotype was 46,XX and chromosomal microarray did not reveal pathogenic variants. Her mother was unaffected, at least no obvious signs of symptom based on the reports of the family, though no formal evaluation was performed.

Clinical information concerning the patient was collected in Shanghai Children’s Medical Center in 2012 (see Table 2). Written consent was obtained from the patient’s parents. 

For whole exome sequencing, genomic DNA was extracted from ethylene diamine tetra acetic acid-treated peripheral blood. Library preparation was performed on the proband with xGen Exome research panel v1.0 (Integrated DNA Technologies Inc, Coralville, Iowa, USA). The captured DNA fragments were subsequently sequenced by HiSeq 4000 (Illumina Inc, San Diego, California, USA). The data were analyzed as previously described (26). The pattern of X-chromosome inactivation in our patient and her mother was evaluated by assays of differential methylation in the genes between the active and the inactive chromosome X based on methylation-specific polymerase chain reaction (PCR) (27).

## Results

The clinical features of the proband are presented in Figure 1A, 1B, 1C, 1D and 1E and Table 2. For comparison with previously reported phenotypes, we reviewed the description of a total of 20 female and 43 male BFLS patients in the literature (Table 2) and summarized the endocrinological presentations (Table 3). Whole exome sequencing revealed a heterozygous nonsense mutation c.673C>T; p.R225X (NM_001015877) of *PHF6 *gene in the proband. Sanger sequencing of the proband and her parents demonstrated that the heterozygous mutation was inherited from her mother. No other variant with clinical significance was identified. Methylation-specific PCR of peripheral blood DNA indicated a highly skewed X-inactivation in the patient (98:2) and in her mother (95:5) (Figure 1F).

## Discussion

BFLS is an X-linked syndrome caused by variants in *PHF6* (7,8). The most prevalent features, as observed in >80% of reported BFLS cases were: ID, delay in walking, delay in speech, coarse facies, dental abnormalities, large ears and finger deformities in females. Additionally, genital anomalies and gynecomastia have been frequently reported in male BFLS patients.

The phenotypic features of our patient largely conform to the description of BFLS based on patients of European ancestry. However, complete deficiency of growth hormone was not reported in previous cases. Our patient’s height was below the 3^rd^ percentile, which has been reported in 14% of female BFLS patients previously (3). She developed edema in the lower extremities after injection of rhGH (before the *PHF6* mutation was identified). Peripheral edema has occurred in 1:100-1:10000 of patients receiving rhGH therapy (28), possibly due to the impact on fluid homeostasis with retention of water and sodium (29). To date, a total of five BFLS patients have been reported to have growth hormone deficiency (Table 3) and two of these presented with multiple pituitary hormone deficiency. The authors reported no improvement of stature after GH treatment (15). Considering the adverse effect in our patient, GH use in this condition may not be helpful and should be administered with caution. This is compounded by recent research showing that *PHF6* mutation may be associated with pediatric leukemia (30).

Genital anomalies were reported in 59% (27/46) of patients. Early literature reported that hypogonadism was caused by hypophyseal dysfunction (1), but recent publications reporting a male patient with low testosterone and elevated LH and FSH, and another patient with abnormal testicular tissue, suggested that both central and gonadal deregulation may occur (23). Review of the literature reveals that the concentration of estradiol was reduced in 2/4 of the female patients and that of testosterone in 12/15 of the male patients. Gonadotrophin concentrations were found to be below reference values in 8/23 patients and hypothyroidism was reported to be present in 6/15 patients, also suggesting that both central and end-organ dysfunction may play a role in BFLS.

As reported in previous studies, hyperpigmentation is common in female BFLS patients with 10 of 13 female patients being hyperpigmented (3,13). Most of these patients were reported to have linear pigmentation in the extremities or individual spots in the armpit (3,13). However in our patient the hyperpigmentation was extensively distributed over the feet and legs. Mosaicism may be the cause for the different presentation in this case. The exact mechanism of hyperpigmentation in BFLS is unknown.

One additional feature of our patient that does not fit the description of classic BFLS is presence of a horseshoe kidney. In an earlier report, clinical phenotypes of BFLS were noted to partially overlap with the Coffin-Siris syndrome (CSS) (12), particularly in infancy among female patients (4). CSS is characterized by ID, typical facial features, hypoplasia/aplasia of the fifth digit of finger/toenail, and organ malformations including horseshoe kidney (4,12). Our patient exhibited many phenotypes overlapping with CSS, including presence of horseshoe kidney. We specifically reviewed the variants of CSS-related genes identifed in our patients, and no pathogenic variant was found. Therefore the similar phenotypes should not be attributed to CSS-related variants. It is well established that *PHF6* interacts with the nucleosome remodeling and deacetylation complex, implicated in chromatin remodeling, and thus functional interaction may exist between *PHF6* and SWI/SNF complex proteins, which are the main factors responsible for CSS (3). This may explain the overlapping features of these two syndromes.

As indicated in Table 2, the penetrance in female carriers is about 44% (21/48). 38/43 of females with *PHF6* mutations had highly skewed X-inactivation, but only 18 of them were affected. Our patient and her mother had the same genotype and similar skewing in X-inactivation. However, their clinical manifestations were quite different, suggesting mosaicism as a contributing factor to the variable expression of the phenotype (12). At the same time, this phenomenon suggests that in obligate carriers of *PHF6* mutations, the level of X-inactivation skewing measured in peripheral blood cells may not be a reliable predictor of the expression of BFLS phenotypes (5).

The limitation of this report is that the manifestation of complete GH deficiency and horseshoe kidney was based on only one patient. Reports of more cases would help to clarify the risk involved in rhGH treatment in this condition.

In conclusion, we report a female with a novel nonsense mutation c.673C>T (p.R225X) of the *PHF6 *gene. The patient exhibited certain features beyond classic BFLS, including horseshoe kidney and complete deficiency of growth hormone. An adverse effect was elicited with GH treatment, suggesting caution in the use of GH in this condition. Both the patient and her unaffected mother had skewing of X-inactivation indicating that X-inactivation assay may not reliably predict the expression of BFLS phenotypes. These clinical and genetic findings may contribute to improve our understanding of BFLS and also aid in the diagnosis and genetic counseling of the condition.

## Figures and Tables

**Table 1:**
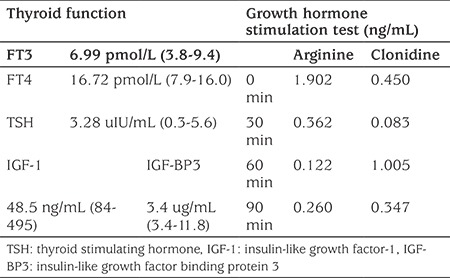
Results of endocrine tests

**Table 2:**
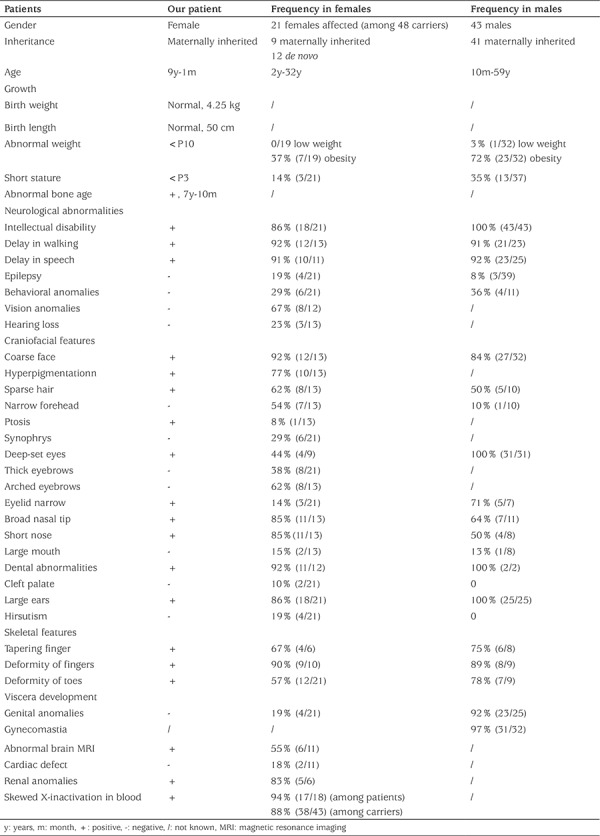
Clinical information on our patient and on reported Borjeson-Forssman-Lehmann syndrome patients

**Table 3:**
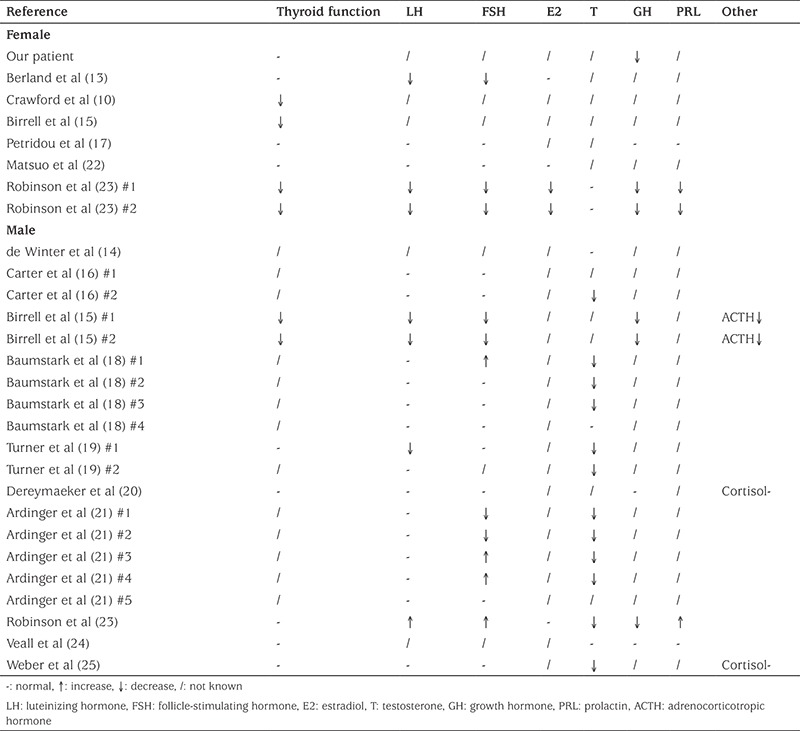
Summary of hormone levels in Borjeson-Forssman-Lehmann syndrome patients

## References

[1] Borjeson M, Forssman H, Lehmann O (1962). An X-linked, recessively inherited syndrome characterized by grave mental deficiency, epilepsy, and endocrine disorder. Acta Med Scand.

[2] Wieczorek D, Bögershausen N, Beleggia F, Steiner-Haldenstätt S, Pohl E, Li Y, Milz E, Martin M, Thiele H, Altmüller J, Alanay Y, Kayserili H, Klein-Hitpass L, Böhringer S, Wollstein A, Albrecht B, Boduroglu K, Caliebe A, Chrzanowska K, Cogulu O, Cristofoli F, Czeschik JC, Devriendt K, Dotti MT, Elcioglu N, Gener B, Goecke TO, Krajewska-Walasek M, Guillén-Navarro E, Hayek J, Houge G, Kilic E, Simsek-Kiper PÖ, López-González V, Kuechler A, Lyonnet S, Mari F, Marozza A, Mathieu Dramard M, Mikat B, Morin G, Morice-Picard F, Ozkinay F, Rauch A, Renieri A, Tinschert S, Utine GE, Vilain C, Vivarelli R, Zweier C, Nürnberg P, Rahmann S, Vermeesch J, Lüdecke HJ, Zeschnigk M, Wollnik B (2013). A comprehensive molecular study on Coffin-Siris and Nicolaides-Baraitser syndromes identifies a broad molecular and clinical spectrum converging on altered chromatin remodeling. Hum Mol Genet.

[3] Zweier C, Kraus C, Brueton L, Cole T, Degenhardt F, Engels H, Gillessen-Kaesbach G, Graul-Neumann L, Horn D, Hoyer J, Just W, Rauch A, Reis A, Wollnik B, Zeschnigk M, Lüdecke HJ, Wieczorek D (2013). A new face of Borjeson-Forssman-Lehmann syndrome? De novo mutations in PHF6 in seven females with a distinct phenotype. J Med Genet.

[4] Zweier C, Rittinger O, Bader I, Berland S, Cole T, Degenhardt F, Di Donato N, Graul-Neumann L, Hoyer J, Lynch SA, Vlasak I, Wieczorek D (2014). Females with de novo aberrations in PHF6. clinical overlap of Borjeson-Forssman-Lehmann with Coffin-Siris syndrome. Am J Med Genet C Semin Med Genet.

[5] Gecz J, Turner G, Nelson J, Partington M (2006). The Börjeson-Forssman-Lehman syndrome (BFLS, MIM #301900). Eur J Hum Genet.

[6] Turner G, Lower KM, White SM, Delatycki M, Lampe AK, Wright M, Smith JC, Kerr B, Schelley S, Hoyme HE, De Vries BB, Kleefstra T, Grompe M, Cox B, Gecz J, Partington M (2004). The clinical picture of the Börjeson-Forssman-Lehmann syndrome in males and heterozygous females with PHF6 mutations. Clin Genet.

[7] Lower KM, Turner G, Kerr BA, Mathews KD, Shaw MA, Gedeon AK, Schelley S, Hoyme HE, White SM, Delatycki MB, Lampe AK, Clayton-Smith J, Stewart H, van Ravenswaay CM, de Vries BB, Cox B, Grompe M, Ross S, Thomas P, Mulley JC, Gécz J (2002). Mutations in PHF6 are associated with Börjeson-Forssman-Lehmann syndrome. Nat Genet.

[8] Mangelsdorf M, Chevrier E, Mustonen A, Picketts DJ (2009). Borjeson-Forssman-Lehmann Syndrome due to a novel plant homeodomain zinc finger mutation in the PHF6 gene. J Child Neurol.

[9] Lower KM, Solders G, Bondeson ML, Nelson J, Brun A, Crawford J, Malm G, Börjeson M, Turner G, Partington M, Gécz J (2004). 1024C> T (R342X) is a recurrent PHF6 mutation also found in the original Börjeson-Forssman-Lehmann syndrome family. Eur J Hum Genet.

[10] Crawford J, Lower KM, Hennekam RC, Van Esch H, Mégarbané A, Lynch SA, Turner G, Gécz J (2006). Mutation screening in Borjeson-Forssman-Lehmann syndrome: identification of a novel de novo PHF6 mutation in a female patient. J Med Genet.

[11] Vallee D, Chevrier E, Graham GE, Lazzaro MA, Lavigne PA, Hunter AG, Picketts DJ (2004). A novel PHF6 mutation results in enhanced exon skipping and mild Börjeson-Forssman-Lehmann syndrome. J Med Genet.

[12] Di Donato N, Isidor B, Lopez Cazaux S, Le Caignec C, Klink B, Kraus C, Schrock E, Hackmann K (2014). Distinct phenotype of PHF6 deletions in females. Eur J Med Genet.

[13] Berland S, Alme K, Brendehaug A, Houge G, Hovland R (2011). PHF6 Deletions May Cause Borjeson-Forssman-Lehmann Syndrome in Females. Mol Syndromol.

[14] de Winter CF, van Dijk F, Stolker JJ, Hennekam RC (2009). Behavioural phenotype in Börjeson-Forssman-Lehmann syndrome. J Intellect Disabil Res.

[15] Birrell G, Lampe A, Richmond S, Bruce SN, Gécz J, Lower K, Wright M, Cheetham TD (2003). Borjeson-Forssman-Lehmann syndrome and multiple pituitary hormone deficiency. J Pediatr Endocrinol Metab.

[16] Carter MT, Picketts DJ, Hunter AG, Graham GE (2009). Further clinical delineation of the Börjeson-Forssman-Lehmann syndrome in patients with PHF6 mutations. Am J Med Genet A.

[17] Petridou M, Kimiskidis V, Deligiannis K, Kazis A (1997). Borjeson-Forssman-Lehmann syndrome: two severely handicapped females in a family. Clin Neurol Neurosurg.

[18] Baumstark A, Lower KM, Sinkus A, Andriuskeviciute I, Jurkeniene L, Gécz J, Just W (2003). Novel PHF6 mutation p.D333del causes Börjeson-Forssman-Lehmann syndrome. J Med Genet.

[19] Turner G, Gedeon A, Mulley J, Sutherland G, Rae J, Power K, Arthur I (1989). Börjeson-Forssman-Lehmann syndrome: clinical manifestations and gene localization to Xq26-27. Am J Med Genet.

[20] Dereymaeker AM, Fryns JP, Hoefnagels M, Heremans G, Marien J, van den Berghe H (1986). The Borjeson-Forssman-Lehmann syndrome. A family study. Clin Genet.

[21] Ardinger HH, Hanson JW, Zellweger HU (1984). Börjeson-Forssman-Lehmann syndrome. further delineation in five cases. Am J Med Genet.

[22] Matsuo K, Murano I, Kajii T (1984). Borjeson-Forssman-Lehmann syndrome in a girl. Jinrui Idengaku Zasshi.

[23] Robinson LK, Jones KL, Culler F, Nyhan WL, Sakati N, Jones KL (1983). The Börjeson-Forssman-Lehmann syndrome. Am J Med Genet.

[24] Veall RM, Brett EM, Rivinus TM, Stephens R (1979). The Börjeson-Forssman-Lehmann syndrome: a new case. J Ment Defic Res.

[25] Weber FT, Frias JL, Julius RL, Felman AH (1978). Primary hypogonadism in the Borjeson-Forssman-Lehmann syndrome. J Med Genet.

[26] Sun Y, Hu G, Liu H, Zhang X, Huang Z, Yan H, Wang L, Fan Y, Gu X, Yu Y (2017). Further delineation of the phenotype of truncating KMT2A mutations: The extended Wiedemann-Steiner syndrome. Am J Med Genet A.

[27] Kubota T, Nonoyama S, Tonoki H, Masuno M, Imaizumi K, Kojima M, Masuno M, Imaizumi K, Kojima M, Wakui K, Shimadzu M, Fukushima Y (1999). A new assay for the analysis of X-chromosome inactivation based on methylation-specific PCR. Hum Genet.

[28] Pfaffle R (2015). Hormone replacement therapy in children: The use of growth hormone and IGF-I. Best Pract Res Clin Endocrinol Metab.

[29] Ho KY, Kelly JJ (1991). Role of growth hormone in fluid homeostasis. Horm Res.

[30] de Rooij JD, van den Heuvel-Eibrink MM, van de Rijdt NK, Verboon LJ, de Haas V, Trka J, Baruchel A, Reinhardt D, Pieters R, Fornerod M, Zwaan CM (2016). PHF6 mutations in paediatric acute myeloid leukaemia. Br J Haematol.

